# Ceramides predict verbal memory performance in coronary artery disease patients undertaking exercise: a prospective cohort pilot study

**DOI:** 10.1186/1471-2318-13-135

**Published:** 2013-12-12

**Authors:** Mahwesh Saleem, Veera V Ratnam Bandaru, Nathan Herrmann, Walter Swardfager, Michelle M Mielke, Paul I Oh, Prathiba Shammi, Alexander Kiss, Norman J Haughey, Randal Rovinski, Krista L Lanctôt

**Affiliations:** 1 Neuropsychopharmacology Research Group, Sunnybrook Health Sciences Centre, Toronto, Ontario M4N 3M5, Canada; 2 Department of Pharmacology and Toxicology, University of Toronto, Toronto, Ontario M5S 1A8, Canada; 3 Department of Neurology, Johns Hopkins University School of Medicine, Baltimore, MD 21231, USA; 4 Department of Psychiatry, University of Toronto, Toronto, Ontario M5A 4L8, Canada; 5 Departments of Neurology and Health Sciences Research, Mayo Clinic, Rochester, MN 55905, USA; 6 Division of Clinical Pharmacology, Sunnybrook Health Sciences Centre, Toronto, Ontario M4N 3M5, Canada; 7 Toronto Rehabilitation Institute, Toronto, Ontario M4G 1R7, Canada; 8 Neuropsychology, Sunnybrook Health Sciences Centre, Toronto, Ontario, Canada; 9 Clinical Epidemiology, Sunnybrook Health Sciences Centre, Toronto, Ontario M4N 3M5, Canada; 10 Department of Psychiatry, Johns Hopkins University School of Medicine, Baltimore, MD 21287-7413, USA

**Keywords:** Ceramides, Memory, Verbal memory, Coronary artery disease, Exercise, Cognitive deficits

## Abstract

**Background:**

Coronary artery disease (CAD) is associated with verbal memory decline, although deterioration may be mitigated in individuals undertaking exercise interventions. Ceramide sphingolipids, suggested to play a role in pathological neurodegeneration, have been associated with the development and progression of CAD but their relationship with cognitive response to exercise has not been assessed. In this study, concentrations of very long chain ceramides (C22:0 and C24:0) were assessed as predictors of changes in verbal memory performance over 1 year in subjects with CAD undertaking cardiac rehabilitation (CR).

**Methods:**

Verbal memory was measured using the California Verbal Learning Test 2^nd^ Ed. (CVLT-II), from which Z-scores were calculated based on age, gender and education matched norms. Baseline plasma C22:0 and C24:0 ceramide concentrations were measured from fasting blood samples using high performance liquid chromatography coupled electrospray ionization tandem mass spectrometry (LC/MS/MS). Repeated measures general linear models were used to determine the association between baseline plasma ceramides and the change in verbal memory performance over 1 year of CR controlling for age and body mass index (BMI).

**Results:**

In patients with CAD (n = 33, mean age = 62 ± 9 years, 84.8% male, years of education = 17 ± 3 years), higher baseline plasma C22:0 (F_1, 29_ = 5.30, p = 0.03) and C24:0 (F_1, 29_ = 4.04, p = 0.05) concentrations significantly predicted less improvement in verbal memory performance over 1 year of CR controlling for age and BMI.

**Conclusions:**

Plasma ceramide concentrations should be further examined as potential predictors of cognitive response to exercise and worse cognitive outcomes in patients with CAD.

**Trial registration:**

NCT01625754

## Background

Coronary artery disease (CAD) is a leading cause of disability and is responsible for approximately 7.3 million deaths worldwide [1]. An under-recognized symptom of particular importance in this population is cognitive impairment, with specific disruptions in multiple cognitive domains [2,3]. In particular, subtle changes in verbal memory performance may be an important marker of cognitive decline in CAD [3]. Cardiovascular risk factors such as obesity, hypertension, and abnormal lipid metabolism have been associated with poorer verbal memory performance [3] and an increased rate of verbal memory decline [2]. For CAD patients, cognitive functioning is a major determinant of quality of life [4] with verbal memory function being especially predictive of mortality [5], physical disability [6], progression to dementia [7] and interference with secondary prevention [8].

Elevated plasma concentrations of the very long chain ceramides C22:0 and C24:0 have been associated with the development of CAD [9] and with greater verbal memory decline and hippocampal volume loss in patients with mild cognitive impairment [10] and incident Alzheimer’s disease in community dwelling elderly women [11]. While ceramides have emerged as promising clinical biomarkers of cognitive decline [10-12], their association with cognitive response to exercise has not been studied.

Exercise is increasingly recognized as a promising intervention to not only improve cardiac outcomes [13] but also to delay verbal memory decline [14,15]; however, effects of fitness and cognitive response to exercise can be heterogeneous [16] indicating a need to explore the mechanisms that may hinder the cognitive benefits of exercise. Proinflammatory [17] and pro-apoptotic [18] properties of ceramide lipid species may impair neural adaptation to exercise and induce neurodegenerative processes [19]. The present study evaluated the relationship between plasma concentrations of C22:0 and C24:0 and change in verbal memory performance in CAD subjects undertaking a 1-year cardiac rehabilitation (CR) program.

## Methods

### Study design

The protocol, including acquisition of informed consent, for this prospective study was approved by the Sunnybrook Health Sciences Centre Research Ethics Board and the University Health Network Research Ethics Board. Patients with CAD were recruited from a CR program as described previously [8] and followed for 1 year. Briefly, the CR program at the Toronto Rehabilitation Institute is comprised of both aerobic and resistance exercise under the supervision of exercise and medical specialists. Following assessment of peak oxygen uptake (VO_2peak_), a measure of fitness, participants attended exercise visits that included an aerobic walk or walk/jog per week for 36 weeks and once per month for the remaining 3 months of the year. Participants were also expected to independently exercise 5 out of 7 days of the week. Participants were followed over the standard 48-week CR protocol. Ceramide assays were performed at baseline. Memory testing was performed at baseline and at 1-year follow-up.

### Subjects

Eligible subjects who provided written informed consent were assessed for inclusion/exclusion criteria. All CR subjects had evidence of CAD (previous hospitalization for acute myocardial infarction, coronary angiographic evidence of ≥ 50% blockage in one or more major coronary artery or prior revascularization). All subjects had dyslipidemia and were being treated with statins. Subjects were excluded based on previously diagnosed neurodegenerative illness including all-cause dementia, active cancer, surgery planned within 12 months, schizophrenia, bipolar affective disorder, and substance abuse. The Mini Mental Status Examination (MMSE) was used to screen for dementia and subjects with MMSE < 24 [20] were excluded.

### Assessments

Verbal memory was assessed using the California Verbal Learning Test 2^nd^ Ed. (CVLT-II), which yields a sensitive measure of long delay free recall (LDFR) of a word list after 20 minutes in addition to measures of verbal learning (recall of a word list over 5 learning trials) and short delay free recall (recall of a word list after 10 minutes) [21]. A trained researcher administered the CVLT-II at a standardized time (0930 hr ± 30 min). A Z-score was computed from age, gender and education matched norms. Z-scores follow a normal distribution with a standard deviation of 1.0; higher Z-scores reflect better performance.

Demographic and clinical characteristics, as well as a detailed medical history including comorbidities independent of CAD were collected from patient interviews. Cardiac medical history, concomitant medications, cardiac health indicators (body mass, height, hyperlipidemia, hypertension, diabetes, waist circumference) and anthropometrics were obtained from patient charts at the Toronto Rehabilitation Institute. Body mass index (BMI) was calculated per standard definition [mass (kg)/(height (m)^2^)].

### Sphingolipid measurements

Fasting blood was drawn at 0900 h ± 30 min, on the same day as the cognitive testing, centrifuged at 1000 g for 10 min at 4°C. Plasma was immediately isolated and stored at −80°C until analysis. Quantification of individual ceramide species was accomplished by high performance liquid chromatography coupled electrospray ionization tandem mass spectrometry (LC/MS/MS) using multiple reaction monitoring as previously described [22]. Ceramide species were assessed as continuous variables in units of counts per second (cps), a commonly used quantification of area under the curve for multiple reaction monitoring assays.

### Statistical analysis

All analyses were performed using SPSS statistical software (version 19.0; IBM, Armonk, NY) and considered significant at a two-tailed p ≤ 0.05. Plasma ceramide measurements were log-transformed and associations between ceramide concentrations and baseline patient characteristics were assessed by Pearson correlations or univariate analyses of variance as appropriate. Repeated measures general linear models were used to determine the association between baseline plasma ceramide concentrations and the change in CVLT-II Z-scores between baseline and follow-up visits. Age and BMI were included as possible confounders *a priori* based on previous analyses and reports in the literature [10,12]. Pearson correlations were used to determine the direction of the associations using change scores over 1 year calculated by subtracting the baseline Z-scores from the follow-up Z-scores.

## Results

Demographics and cardiac risk factors are reported in Table 1 (n = 33). Mean age was associated with baseline C24:0 (r = −0.38, p = 0.03) but not C22:0 (r = −0.24, p = 0.18) concentrations. There were no other significant associations between baseline ceramide concentrations and sociodemographic characteristics, cardiac risk factors, CAD severity, medical comorbidities, cardiopulmonary fitness parameters or concomitant medications used.

**Table 1:** Demographics and clinical characteristics of subjects (n = 33) and associations with C22:0 and C24:0 concentrations

	**CAD (n = 33) Mean ± SD or % (n)**	**Statistic (F or r)**	C22:0 P-value (p ≤ 0.05)*	**Statistic (F or r)**	C24:0 P-value (p ≤ 0.05)*
**Sociodemographics**					
Age (yrs)	62 ± 9	r = −0.24	0.18	r = −0.38	0.03*
Sex (% male)	84.8 (28)	F = 0.16	0.69	F = 0.12	0.74
Ethnicity (% Caucasian)	90.9 (30)	F = 0.81	0.38	F = 0.28	0.60
Marital status (% married)	75.8 (25)	F = 2.56	0.12	F = 2.00	0.17
Employed (% employed)	48.5 (16)	F = 0.03	0.86	F = 0.07	0.79
Years of education (yrs)	17 ± 3	r = −0.01	0.97	0.09	0.62
**Body composition**					
Body mass index (kg/m^2^)	28.2 ± 3.6	r = 0.27	0.13	r = 0.27	0.13
Body fat (%)	28.3 ± 7.4	r = 0.24	0.19	r = 0.19	0.29
Body mass (pounds)	185.1 ± 27.3	r = 0.22	0.22	r = 0.27	0.13
Waist circumference (cm)	99.6 ± 9.7	r = 0.08	0.68	r = 0.08	0.67
**CAD severity**					
Cumulative stenosis (%)	132.9 ± 45.9	r = 0.05	0.78	r = 0.06	0.73
Number of vessels stenosed	2 ± 1	r = 0.02	0.92	r = 0.11	0.54
Time since acute coronary event (wks)	28.7 ± 76.7	r = 0.09	0.61	r = 0.11	0.53
**Cardiac history % (n)**					
Myocardial infarction	54.5 (18)	F = 1.38	0.25	F = 3.40	0.08
Coronary artery bypass graft surgery	39.4 (13)	F = 0.97	0.33	F = 1.79	0.19
Stent	57.6 (19)	F = 1.20	0.28	F = 3.94	0.06
Angina	27.3 (9)	F = 0.55	0.46	F = 0.89	0.35
Hypertension	57.6 (19)	F = 0.65	0.43	F = 0.30	0.59
**Comorbidities % (n)**					
Diabetes	15.2 (5)	F = 0.85	0.37	F = 1.09	0.30
Depression	36.4 (12)	F = 1.48	0.23	F = 1.19	0.28
Hypercholesterolemia	100 (33)	-	-	-	-
**Cardiopulmonary fitness parameters**					
Maximum heart rate (bpm)	124.3 ± 19.6	r = 0.13	0.46	r = 0.18	0.33
Maximum systolic blood pressure (mm Hg)	181.6 ± 28.3	r = 0.15	0.40	r = 0.06	0.73
Maximum diastolic blood pressure (mm Hg)	82.4 ± 11.6	r = −0.004	0.98	r = 0.01	0.94
VO_2_ peak (ml/kg/min)	20.7 ± 5.3	r = −0.13	0.49	r = −0.10	0.60
**Medications % (n)**					
B-blocker	81.8 (27)	F = 0.17	0.68	F = 0.01	0.93
Diuretic	21.2 (7)	F = 0.90	0.35	F = 0.15	0.70
Anti-hypertensive	57.6 (19)	F = 0.67	0.42	F = 0.13	0.72
Ca^2+^ channel blocker	12.1 (4)	F = 0.11	0.74	F = 0.35	0.56
Antidiabetic	9.1 (3)	F = 0.65	0.43	F = 0.25	0.62
Statin	100 (33)	-	-	-	-
Anxiolytic	6.1 (2)	F = 0.84	0.37	F = 0.69	0.41
Antidepressant	6.1 (2)	F = 0.30	0.59	F = 0.41	0.53

*two-tailed significance in one-way ANOVA or Pearson correlations.

**Figure 1:**
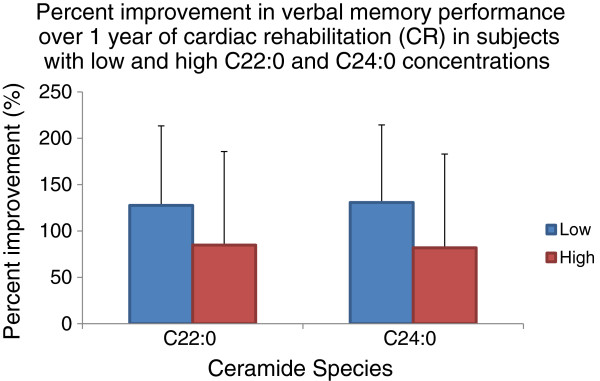
**Percent improvement in verbal memory performance over CR in low (bottom 50%) and high (top 50%) ceramide groups.** In a repeated measures model, significant within subjects effects (F_1,29_ = 5.30, p = 0.03; left panel) indicated less improvement in verbal memory performance over 1 year in subjects with high C22:0 concentrations (red) compared those with low C22:0 concentrations (blue). Less improvement in verbal memory performance over 1 year (F_1,29_ = 4.04, p = 0.05; right panel) was also found in subjects with high C24:0 concentrations (red) compared to those with low C24:0 concentrations (blue). No significant between-subjects effects were found.

Median plasma C22:0 concentrations at baseline were 1.53 × 10^6^ cps (interquartile range = 1.06 × 10^6^ - 1.93 × 10^6^) and C24:0 concentrations were 7.81 × 10^6^ (interquartile range = 5.94 × 10^6^ - 1.02 × 10^7^). Mean LDFR Z-score increased from 0.06 ± 1.01 at baseline to 0.94 ± 0.97 at follow-up after 1 year. In repeated measures analyses controlling for age and BMI, significant ceramides × time interactions were found within subjects; higher baseline log C22:0 (F_1, 29_ = 5.30, p = 0.03) and C24:0 (F_1, 29_ = 4.04, p = 0.05) concentrations were significantly predictive of less improvement in verbal memory performance. As shown in Figure 1, subjects with high plasma C22:0 and C24:0 concentrations (top 50%) had 34% and 37% less improvement in verbal memory performance compared to those with low plasma C22:0 and C24:0 concentrations (bottom 50%) respectively. Between-subjects comparisons showed no associations between LDFR Z-scores and baseline concentrations of C22:0 (F_1, 29_ = 1.18, p = 0.29) or C24:0 (F_1, 29_ = 0.27, p = 0.61).

The associations between C22:0 (F_1, 29_ = 4.67, p = 0.04) and C24:0 (F_1, 29_ = 4.91, p = 0.04) and less improvement in LDFR scores remained significant in a post-hoc repeated measures model controlling for baseline VO_2peak_ and age.

## Discussion

This is the first study to assess the relationship between very long chain ceramide species measured in the blood and memory performance in patients with CAD. This study demonstrates an association between higher baseline plasma ceramide concentrations and less improvement in verbal memory performance in patients with CAD participating in CR. We observed an inverse correlation between plasma ceramide concentrations and age among individuals with CAD, which is in line with Gorska et al. [23]. However, many other studies, mostly in animal models, suggest that ceramides increase with age (please see review of Cutler & Mattson [24]). The relationships of ceramides to age are complex and appear to depend on the species of ceramide and site of sampling (peripheral versus brain versus cerebrospinal fluid). As such, the exact relationships between age and ceramide concentrations remain to be determined. Epidemiological studies with large sample sizes examining ceramide levels in the population, and factors that affect them (e.g., gender, disease states) will be needed to address this question. Baseline concentrations of plasma ceramides in this study were similar to other reports that have used LC/MS/MS with quantitative measures of analyte area [12,25]. Baseline participant memory scores were in the non-impaired range and consistent with other studies assessing memory impairment in healthy elderly [12] and those with CAD [8,26]; however, even subtle deficits in verbal memory performance are associated with poorer clinical outcomes in patients with CAD [8].

Overall, participants experienced an improvement in verbal memory performance over the course of the 1-year CR program, which is consistent with a large body of evidence showing improvements in cognitive performance due to physical exercise in the elderly [15,27]. However, patients with higher baseline plasma C22:0 and C24:0 concentrations showed less improvement when controlling for age and BMI compared to those with lower baseline plasma ceramide concentrations. The magnitude of the estimate suggests that a 1 standard deviation higher baseline C22:0 concentration predicted less improvement on the CVLT-II by approximately 1–2 words, which is equivalent to at least 8 years of cognitive aging.

These findings are consistent with previous findings in 98 elderly community dwelling women where higher concentrations of serum very long chain ceramides including C24:0 were significantly associated with an increased risk of incident verbal memory impairment and Alzheimer’s disease over 9 years [11,12]. Similar trends were observed between higher baseline plasma concentrations (C22:0 and C24:0) and decline in verbal memory performance on the CVLT in a prospective study of subjects with mild cognitive impairment. In that study, higher plasma concentrations of C22:0 and C24:0 at baseline also predicted hippocampal volume loss over 1 year [10]. Consistent with the between-subjects findings in the present study, no cross-sectional relationships between plasma ceramides and verbal memory test scores were identified in those studies. Taken together, these findings suggest that high ceramide levels in the blood may be relevant to memory changes over time in patients with CAD despite exercise.

While neural adaptations to exercise are incompletely understood, they are associated with markers of neurogenesis and angiogenesis in patients with CAD [28]. This may be particularly relevant in the hippocampus [29] since physical activity can reverse loss of hippocampal volume, a correlate of memory performance, associated with aging [14]. It could be hypothesized that ceramide mediated formation of reactive oxygen and nitrogen species and resulting oxidative damage in the hippocampus may interfere with neural adaptation to exercise and the suggested involvement of ceramides in apoptotic cascades leading to brain cell death [30] may contribute to neurodegeneration underlying cognitive decline. In addition, ceramide transporting proteins (CERTs) have been recently identified in the blood and shown to interact with proteins associated with dementia pathology such as serum amyloid P component [31,32]. Thus, a variety of mechanisms may relate ceramide concentrations to cognitive decline.

The present findings may also offer preliminary indications of potential therapeutic interventions. Both C22:0 and C24:0 are synthesized by ceramide synthase 2, which has a sphingosine-1-phosphate (S1P) receptor like motif and can be competitively inhibited by fingolimod (fTY720), a first-in-class S1P analogue targeting 4 of the 5 S1P receptor subtypes. Fingolimod has been recently approved by the American Food and Drug Administration for the treatment of multiple sclerosis [33]. Its role in modulating ceramide synthesis and signaling [34] provides proof of concept that ceramide synthesis is a possible drug target. Specifically, inhibiting ceramide synthase 2 may be useful in modulating ceramide synthesis and signaling [34] in CAD patients who show aberrant ceramide metabolism and verbal memory decline. Recently, aerobic exercise was shown to decrease ceramides in the skeletal muscle of obese individuals [35] suggesting that future studies in this population would provide a unique opportunity to assess exercise as an effective intervention for the modulation of aberrant sphingolipid metabolism and preservation of cognitive performance.

This pilot study was limited by a small sample size, which reduced the number of potential confounders that could be controlled for; however, the associations found were robust to adjustment with age, BMI and VO_2peak_. Also, the change in plasma ceramide concentrations was not assessed but changes in ceramide concentrations might have offered further insight into whether exercise can modulate ceramide concentrations. Practice effects may have contributed to the overall improvement in verbal memory; however, a one-year interval between testing would be expected to minimize such effects on tests of verbal memory [36]. While the results of this study may not be generalizable to all patients with CAD undergoing CR, the sample characteristics are similar to a much larger group of CAD patients undergoing CR described previously [37]. Potential referral bias and patients undertaking different exercise regimes might further limit the generalizability of the results. Future prospective longitudinal studies with adequate power are needed to explore the association between plasma ceramides and memory performance and change in VO_2peak_ in subjects with CAD. In addition, the sensitivity and specificity of C22:0 and C24:0 as possible plasma biomarkers of cognitive response in CAD should be assessed in future studies; however, C22:0 and C24:0 may be particularly important as they have been prospectively associated with dementia [11].

## Conclusions

Baseline C22:0 and C24:0 concentrations were significantly associated with change in verbal memory performance over 1 year of CR with higher ceramide concentrations predicting less improvement. These preliminary findings suggest that ceramides and other sphingolipid species should be explored as prospective cognitive biomarkers in patients with CAD. Future studies are needed to clarify the association between sphingolipid metabolism and cognitive changes in this and other populations.

## Competing interests

The authors declare that they have no competing interests.

## Authors’ contributions

MS recruited and interviewed study participants, managed and analyzed the data, prepared the first draft of the manuscript and was involved in the revision of the manuscript. VB carried out the plasma ceramide analyses. NH was involved with the design of the trial, overseeing data analysis and the preparation and revision of the manuscript. WS recruited and interviewed study participants and was involved in the preparation and revision of the manuscript. MM was involved in data analysis and the preparation and revision of the manuscript. PO was involved with the design of the trial, was responsible for overseeing recruitment at Toronto Rehabilitation Institute and helped revised the manuscript. PS was involved with the training of students administering cognitive testing and helped revise the manuscript. AK was involved with the statistical approach adopted for this study. NJH oversaw the ceramide analyses and helped revised the manuscript. RR was involved in the preparation and revision of the manuscript. KL was involved with the design of the trial, overseeing data analysis and the preparation and revision of the manuscript. All authors read and approved the final manuscript.

## Pre-publication history

The pre-publication history for this paper can be accessed here:

http://www.biomedcentral.com/1471-2318/13/135/prepub
